# The reproductive performance of the Mupli beetle, *Luprops tristis* , in relation to leaf age of the para rubber tree, *Hevea brasiliensis*

**DOI:** 10.1093/jis/14.1.12

**Published:** 2014-01-01

**Authors:** T. K. Sabu, P. M. Nirdev, P. Aswathi

**Affiliations:** Post Graduate and Research Department of Zoology, St. Joseph's College, Devagiri, Calicut- 673 008, Kerala, India

**Keywords:** fecundity, leaf age performance, leaf nutrient resorption, leaf substrate quality, survival

## Abstract

An analysis of host plant leaf age preferences and phenology studies led to the predictions that tender rubber plant leaves are essential for the completion of the life cycle of the Mupli beetle, *Luprops tristis* Fabricius (Coleoptera: Tenebrionidae) and that low tender leaf availability during the post-dormancy stage will limit the beetle population. Analyses of the effects of feeding the beetles leaves of various ages, nitrogen (N) content, and moisture content on fecundity and the duration of post-dormancy survival were carried out. The results showed that tender leaf availability during the post-dormancy phase of *L. tristis* is a critical factor that determines the survival of *L. tristis* adults and the subsequent generation. The control of powdery mildew ( *Odium hevea* ) disease-mediated premature leaf fall in rubber plantations may regulate the beetle population. A peak in fecundity during the early phase of post-dormancy is proposed as an adaptive mechanism of *L. tristis* to synchronize egg production and feeding with tender leaf availability in rubber plantations. Variations in nutrient levels and moisture content between deciduous rubber tree leaves of different ages are attributed to the leaf nutrient resorption mechanism of senescing leaves. These results established that tender leaves with high N and moisture levels are essential for post-dormancy survival and that N influences fecundity. The results of the experiments could aid decision making regarding the population management and control of *L. tristis* in rubber plantations.

## Introduction

Massive seasonal invasions of the Mupli beetle, *Luprops tristis* (Fabricius) (Coleoptera: Tenebrionidae), cause various problems. These beetles enter residential buildings following summer showers, are nocturnal, are attracted to light, produce allergenic defensive secretions, and go dormant for 8–9 months, making them an extreme nuisance in the rubber plantation belts of southern India ( Sabu et al. 2008 ; Sabu and Vinod 2009a , b). Their very high abundance, concealment in rubber plantation litter layers, aggregation in residential buildings, and lack of natural enemies ( Aswathi and Sabu 2011 ) make controlling them with conventional methods unfeasible. An analysis of their host plant preferences and habits revealed that plantation litter stands of rubber trees, *Hevea brasiliensis* (Willdenow ex Adrien De Jussieu) Müller Argoviensis 1865 (Malpighiales: Euphorbiaceae), are the breeding and feeding habitats, and prematurely fallen tender rubber leaves are the preferred food source ( Sabu and Vinod 2009a , b; Sabu et al. 2012 ). The breeding phase of the post-dormancy beetles is perfectly synchronized with the annual leaf shedding and sprouting of new rubber plant leaves during the pre-summer period ( Sabu and Vinod 2009b ), and beetles of certain developmental stages (eggs, larval instars, pupae, and teneral adults) peak at the premature fall of the tender leaves (Vinod and Sabu 2009). These findings indicate that tender leaves are important for the completion of the life cycle of *L. tristis* and that the control of tender leaf availability will limit the beetle population ( Sabu and Vinod 2009b ). An empirical analysis of how the lack of tender leaves will affect the reproductive performance and survival of post-dormancy beetles was undertaken as the primary objective of the present study.

The observation that *L. tristis* is attracted to and prefers to feed on tender leaves necessitates an analysis of the nutritional quality of leaves of various ages. There is widespread evidence from herbivorous insects that age-related variation in leaf nutrient quality, especially in nitrogen (N) and moisture levels, affects insect performance and that tender leaf availability is a major factor in determining the most suitable periods for larval development and for the optimal reproductive capabilities of adults ( Schroeder 1986 ; Awmack and Leather 2002; Haukioja 2003 ; Riipi et al. 2004 ; Ruusila et al. 2005 ; Van Asch and Visser 2007 ). However, no data exist on the age-related variations of rubber leaf quality. Hence, age-related variations in N and moisture levels of rubber leaves of various ages were determined, and their influence on the reproductive performance and the survival of *L. tristis* beetles was assessed. These experiments tested if the prevention of premature leaf fall in rubber plantations, a practice almost abandoned in monoculture rubber plantations due to high labor costs, is likely to enable the control of the beetle populations. It is likely to be welcomed by farmers because it is environmentally friendly and because preventing premature leaf fall would lead to higher latex production. We are unaware whether the levels of the major leaf nutrients, sodium (Na), potassium (K), calcium (Ca), and magnesium (Mg), have any role in the selection of rubber litter by the beetles. Hence, in addition to N and moisture content, Na, P, Ca, and Mg leaf levels ( Perry 1994 ; Lal et al. 2001 ) were also estimated in leaves of various ages.

## Materials and Methods

### Collection of beetles and the experimental set-up

*L. tristis* pupae were collected from the rubber tree plantation ( *H. brasiliensis* clone RRII 105) near the Devagiri college campus located at Calicut (11° 15′ N, 75° 48′ E), in the Kerala state of India in March, 2009. Teneral adults were transferred into two large circular clay vessels (13 x 35 cm) that were capped with a nylon mesh net and placed in an environmental chamber (Yorco, http://www.yorco.com ) at 70% relative humidity and 33˚ C (representing the average temperature and humidity in the rubber plantation litter). They were fed a mixture of wilted tender, senescent, and dry rubber tree leaves. To simulate the onset of summer showers starting in the last week of April, rubber leaves were no longer provided, and water was sprayed using a mist sprayer to induce dormancy. A wooden box (15 x 7 x 3 cm) was provided as the dormancy shelter for the beetles (see Sabu et al. 2008 for details).

The experiment started in the last week of December, 2009 when the beetles started showing signs of arousal from dormancy. The beetles were sexed following the sternal-notch method ( Vinod et al. 2008 ). Three leaf ages were tested: wilted tender leaves, yellow-brown senescent leaves, and dry leaves. Each replicate comprised one male-female pair in a small clay vessel (8 x 5 cm) covered with nylon mesh and kept in the environmental chamber. A small, moist piece of cotton placed on the net served as a source of water, and the excreta were removed on a daily basis. The eggs produced were counted and transferred into sterile plastic vials (5.5 x 4.5 cm) using a moist fine hair brush. For each pair, the mating duration, the frequency of oviposition, and the fecundity were recorded. Ten replicates for each leaf age were maintained; thus, a total of 30 beetle pairs were analyzed. A parallel stock of 10 pairs was maintained on each leaf age to replace individuals lost by mortality during the intermediate stages of the experiment. Mortality was estimated as the number of days taken to reach 25, 50, and 100% mortality. Replacement beetles were used only for estimations of fecundity.

The 50 eggs laid during the first 24 hours of oviposition were transferred to Petri dishes (9 x 1.5 cm) in the environmental chamber and monitored at six hour intervals. The number of eggs hatched and the duration of egg development were recorded. Neonate larvae that hatched within a six hour period were transferred to labeled plastic vials (5.5 x 4.5 cm) with a moist, fine hair brush. The vials were covered with fine cotton cloth until the emergence of the 3 ^rd^ instar larvae to prevent the escape of small larval instars and thereafter with nylon mesh. Ten larvae per container were maintained to follow the development of successive larval instars as well as to avoid the inhibitory effect of crowding on larval development in tenebrionids ( Tschinkel and Wilson 1971 ). Larval instars and adults were fed sliced tender rubber leaves. The durations of the larval and pupal stages were recorded.

### Leaf collection

Leaves were collected from randomly selected trees from the same rubber plantation of uniform age raised from a single clone. Leaves of particular ages were not available from an individual tree for the entire course of the experiment, and hence leaves from several trees were used. By pooling leaves from several plants from the same plantation of the same clone and age, we hoped to obtain a fair estimate of nutrient concentrations. Tender leaves are distinctly different in color and size from mature leaves, which are small, brown, and smooth, and they were readily available during the early stages of foliage flush. To meet the requirement for tender leaves towards the late phase of the study, twigs of randomly selected trees were broken, and freshly sprouted leaves were collected from them. Senescent yellow-brown leaves were removed by gently flicking the leaves from the trees. Freshly fallen dry leaves that were brown-yellow were handpicked directly from the upper litter layers. Senescent and dry leaves were collected by tracking the trees that shed leaves late. Subsamples of the leaves collected for feeding the beetles were used for the chemical and moisture analyses.

### Leaf nutrient quality estimation

Moisture content was determined by measuring the fresh weight (FW) of the leaves (to the nearest 0.001 g), drying them in paper envelopes at ambient temperature for three weeks, and re-weighing them (DW). Moisture content was calculated as (FW - DW)/FW ( Nahrung et al. 2009 ). Following the moisture analysis, the dried leaf samples from each two weeks period were pooled, oven-dried (40 ^o^ C for 3 days), ground into powder with a blender, and used for the estimations of nutrient content. Nitrogen content was determined following the Kjeldahl method ( Jackson 1973 ). The estimation of Na, K, Ca, and Mg levels was carried out following the Wet oxidation method ( Jackson 1958 ; Adler and Wilcox 1985 ) using an atomic absorption spectrophotometer (Varian AA 240 FS, Varian Medical Systems, http://www.varian.com ), and P levels were determined by the Vanado molybdate method ( Jackson 1973 ).

### Statistical analysis

Ten replicates of each condition (tender, senescent, and dry leaves) were maintained. A preliminary analysis of the distribution of the data for each parameter was done with the Jarque-Bera test. The moisture content percentages were arcsine square roottransformed prior to statistical analysis. Significance levels of variation in the post-dormancy life spans of the beetles fed leaves of different ages and nutrient parameters (N, Na, K, Ca, Mg, P, and moisture content) were analyzed with oneway ANOVA tests followed by pairwise comparisons with Tukey tests. Variations in fecundity during different phases of post-dormancy were analyzed with the Kruskal Wallis test followed by pairwise comparisons with Mann-Whitney tests ( Weiss 2007 ). The influence of N and moisture content on biweekly fecundity of beetles fed tender leaves and on the post dormancy lifespan of the beetles fed leaves of various ages was examined with multiple regression analysis. In this analysis, the qualitative variable (leaf age) was kept constant and treated as a categorical (dummy) variable. The relationship between N and moisture content was analyzed with the Pearson correlation test to explain the multicollinear relationship between the variables. The leaf minerals (peripheral variables) were excluded from the multiple regression analysis, as they lead to multicollinearity among the variables ( Graham 2003 ; Gujarati 2011 ). The significance levels of all analyses were *p* < 0.05. Minitab 16 Academic software for Windows ( Minitab 2010 ) was used for all statistical analyses.

## Results

Beetles fed tender leaves entered into the reproductive phase, produced eggs, and survived for 135.55 ± 45.81 days, while those fed senescent and dry leaves lived for 28.25 ± 12.43 and 21.6 ± 10.36 days, respectively and did not produce eggs. The preoviposition period for beetles fed tender leaves was 13.9 ± 2.02 days, fecundity was 60.5 ± 40.23 eggs, egg laying events lasted for six months, and egg laying intervals were 6.9 ± 3.31 days.

**Figure 1.**
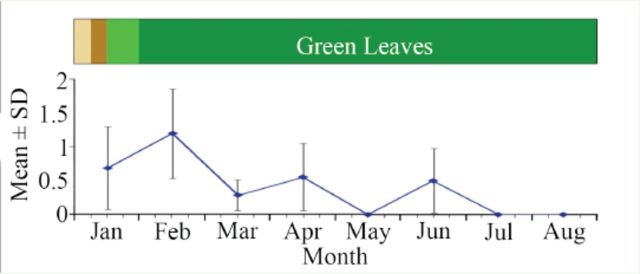
Fecundity* (mean ± SD) of post-dormancy *Luprops tristis* fed exclusively tender rubber leaves in relation to the phenology of *Hevea brasiliensis* leaf shedding (*Log transformed). The tan, brown, and light green boxes indicate falling leaves, sprouting, and light green leaves, respectively. High quality figures are available online.

Two phases, an initial phase of four months of fecundity and an intervening one month eggless period in the 5 ^th^ month, were distinct. The highest fecundity was recorded in the 2 ^nd^ month of the post-dormancy phase ( Figure 1 and Table 3 ). In total, 94% hatching was recorded. The duration of the egg incubation period was 3.43 ± 0.47 days, the larval instar phase lasted 33.74 ± 0.35 days, and the pupal phase lasted 3.08 ± 0.08 days **.**

**Table 3.**
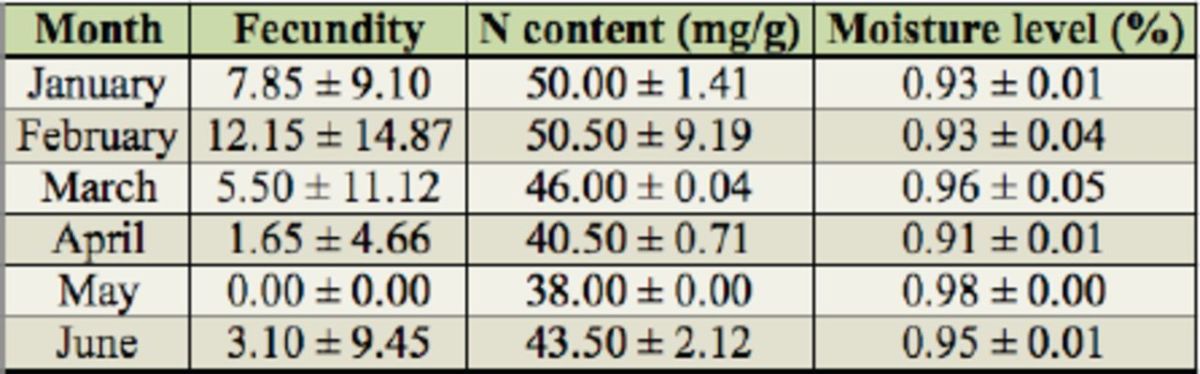
Monthly variation in fecundity of pre-dormancy *L uprops tristis* in relation to the nitrogen level and moisture content of the tender leaves.

**Figure 2.**
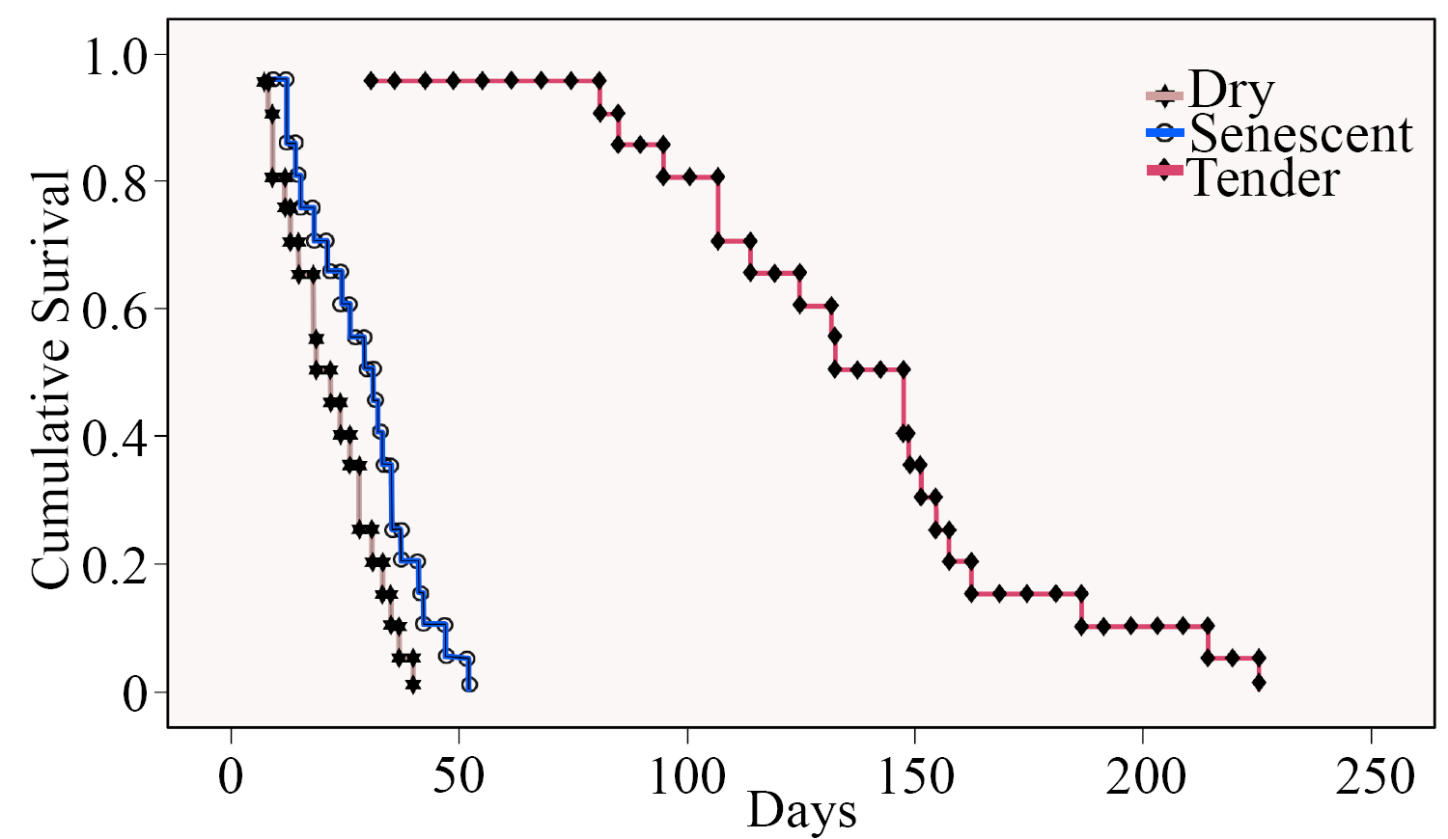
Kaplan Meier survival curve of *Luprops tristis* fed tender, senescent, and dry rubber leaves. High quality figures are available online.

Differences in the post-dormancy life span were distinct among the three cultures *(F =* 108.77, DF = 2, *p* < 0.05). Beetles reared on dry and senescent leaves had similar life spans *(p* < 0.05), whereas those fed tender leaves had a significantly longer life span *(p* > 0.05). The times until the post-dormancy beetles reached 100% mortality were 231 days when fed tender leaves, 56 days when fed senescent leaves, and 42 days when fed dry leaves ( Table 1 and Figure 2 ).

**Table 1.**
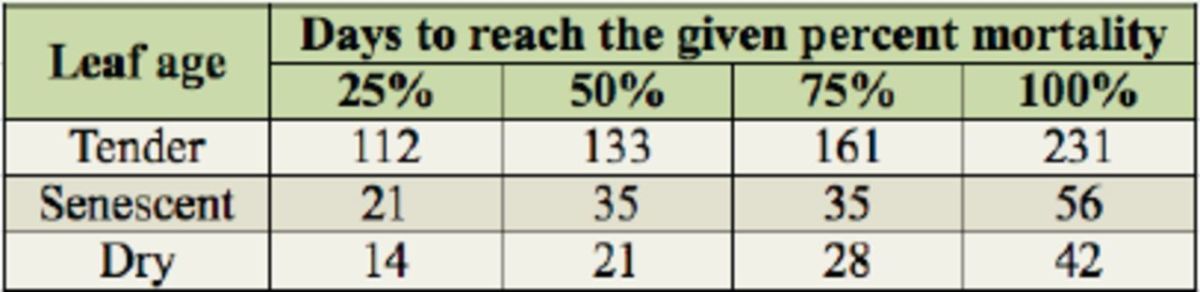
Time (days) for post-dormancy *Luprops tristis* fed tender, senescent, and dry rubber leaves to reach 25, 50, 75, and 100% mortality.

Higher N content was recorded for tender leaves than senescent and dry leaves, and no variation was observed in the levels of N between senescent and dry leaves. Moisture content varied between leaf ages and was highest in tender leaves and lowest in dry leaves. Variation in the levels of the major leaf nutrients were noted, with P and K being highest in tender leaves, Ca being highest in dry leaves, and Na and Mg being highest in senescent and dry leaves ( Table 2 ). Multiple regression analysis revealed a significant influence of the N level and moisture content on the lifespan of the post-dormancy beetles *(p* < 0.05; *F* = 55.39; R ^2^ = 0.89). Multiple regression analysis of the N and moisture leaf content on the fecundity of the beetles revealed a significant influence of N ( *p* ≤ 0.05; T = 2.75; *F* = 41.73; R ^2^ = 0.90) and no influence of moisture ( *p* ≥ 0.05; T = 0.79; *F* = 41.73; R ^2^ = 0.90) on fecundity. Pearson correlation analysis revealed a high correlation between N and moisture content (r = -0.99; *p* ≤ 0.05).

**Table 2.**
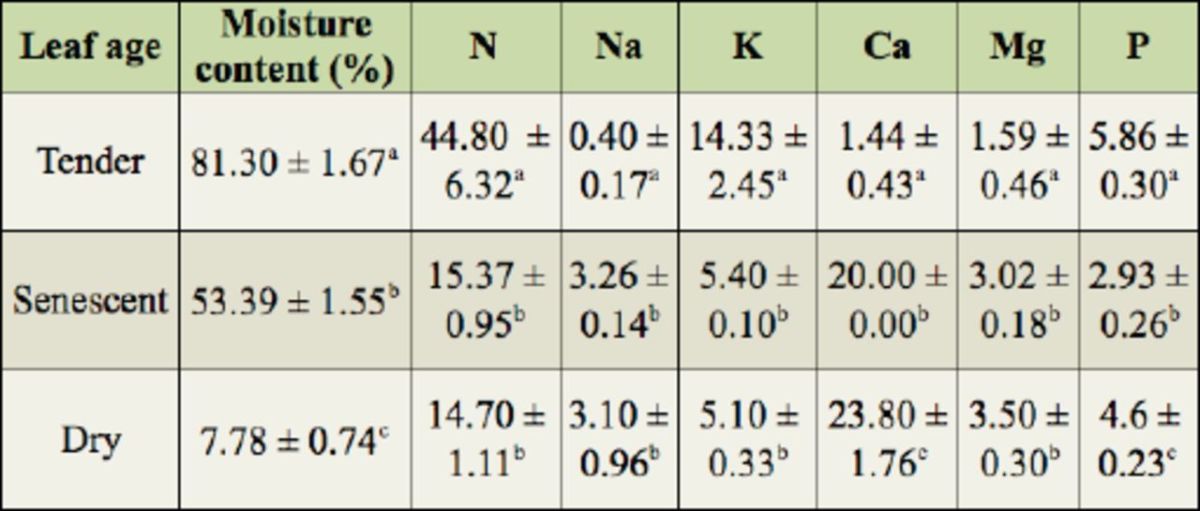
Moisture content (%) and amounts of elements (mg/g) in leaves of the rubber tr ee, *Hevea brasiliensis,* of different ages. The different superscri pt letters within each column indicate means that differ significantly by a Student’s *t* -test *(p* < 0.05).

## Discussion

### Reproduction and survival of post-dormancy *L. tristis* fed leaves of various ages

Post-dormancy *L. tristis* beetles fed senescent and dry rubber leaves exhibited significantly greater mortality and a failure to reproduce compared with those fed tender leaves, demonstrating that tender rubber leaves are essential for *L. tristis* to complete its life cycle. Earlier work on the link between the phenology of the rubber tree and the *L. tristis* life cycle revealed that the high abundance of *L. tristis* in rubber plantations is related to the advantages gained from feeding prematurely fallen tender leaves. Premature leaf fall is caused primarily by powdery mildew ( *Odium hevea* ) and *Corynespora cassiicola* . Hence, it was suggested that the control of the premature leaf fall in rubber plantations may enable control of the pest ( Sabu and Vinod 2009b ; Vinod and Sabu 2009; Sabu et al. 2012 ). The present study provides empirical evidence supporting these earlier predictions. Additionally, the inability of post-dormancy *L. tristis* to survive on senescent and dry leaves beyond 3–4 weeks suggests that reproduction depends on the availability of leaves from the premature leaf fall mediated by powdery mildew disease and not on those from the leaf fall associated with *Corynespora* that occurs 3–4 months later. Hence, the control of the seasonal premature leaf fall in rubber plantations caused by powdery mildew soon after leaf sprouting by spraying fungicides may enable control of this pest. These findings have great practical significance, as they reveal a strategy to tackle this pest that is otherwise not practically feasible either with beetle-directed pesticides or with natural enemies ( Aswathi and Sabu 2011 ). Furthermore, it confirms that tender leaf availability is a major limiting factor regulating the life cycle of *L. tristis* in the moist south Western Ghats in addition to rainfall ( Vinod and Sabu 2010 ). Since tender leaf resource availability is limited to the pre-summer period in monoculture rubber plantation belts, *L. tristis* would remain univoltine in the region.

### The survival of post-dormancy *L. tristis* on dry leaves and its implications

Although post-dormancy *L. tristis* could not enter the reproductive phase, its ability to survive on dry leaves for 3–4 weeks indicates its remarkable potential to survive until leaf sprouting and subsequent tender leaf fall. This could be a strategy to counteract the high mortality experienced during the last phase of dormancy ( Sabu et al. 2008 ), which if continued could lead to death of the entire post-dormancy beetle population and prevent the production of the next generation. One quarter of the beetles perish during the 9 month dormancy period (see Sabu et al. 2008 for details). The present record of 75% mortality of post-dormancy *L. tristis* fed dry leaves within 3-4 weeks indicates that only one-quarter of post-dormancy *L. tristis* returning to rubber plantations could survive and enter the reproductive phase upon the return of tender leaves. The high survival rate of post-dormancy *L. tristis* fed tender leaves suggests that the availability of tender leaves of other rubber clones in the RI 115 plantation (other host plants are unlikely in monoculture rubber plantations) would lead to higher survival rates for the post-dormancy *L. tristis* and a rise in population. These findings provide an answer to the questions raised in earlier studies ( Sabu et al. 2007 ; Sabu and Vinod 2009a ) on whether the post-dormancy *L. tristis* that return to the plantations could survive on dry leaves during the initial phase of leaf fall in rubber plantations. They indicate perfect synchronization of the life cycle of the beetle with host plant phenology at two occasions— first at the time of post-dormancy return and the annual leaf shedding by rubber trees and later at the time of entry into the breeding phase and the premature leaf fall in rubber plantations ( Sabu and Vinod 2009a ).

Leaf age-related variations in mortality indicate that if annual leaf shedding and tender leaf availability are delayed, one could expect high mortality of post-dormancy *L. tristis* . Conversely, if the annual leaf shedding starts prematurely due to the early cessation of the monsoons and the onset of summer conditions, there would be low mortality and larger populations. Such variations in the annual leaf shedding and tender leaf availability could have caused the variation in the abundance of the *L. tristis* population and the intensity of infestation during certain years. Monitoring the variations in leaf shedding would enable the predictions of the severity of infestation necessary to initiate precautionary measures to limit the intensity of home invasions.

### The high fecundity and prolonged post-dormancy phase of *L. tristis* fed tender leaves

The fecundity of beetles fed exclusively tender leaves in laboratory conditions was higher than the fecundity in natural conditions ( Sabu et al. 2008 ). Hence, upon premature leaf fall, the duration of the post-dormancy phase and fecundity will increase (60.5 ± 40.24 eggs in this study under ideal conditions, in contrast to 30.6 ± 13.92 eggs under natural conditions; Sabu et al. 2008 ) leading to larger populations and severe beetle aggregation. However, these conditions lead to the emergence of teneral adults with less time for food reserve accumulation and low survival chances during dormancy ( Sabu et al. 2008 ). Currently, the RR 115 rubber clone with early leaf fall and leaf sprouting during January is being replaced by the RR 414, RR 424, and RR 430 rubber clones, which display delayed leaf shedding and leaf sprouting during February. The combined effect of the late leaf sprouting of the new clones and the early leaf shedding of the old RR 115 clones will lead to a prolonged period of tender leaf availability until the complete replacement occurs over a 10–15 year period. Hence, a further rise in the *L. tristis* population in this region is predicted.

A decline in fecundity towards the late phase of post-dormancy even when tender leaves are available indicates that fecundity variation during the post-dormancy *L. tristis* phase cannot be attributed to the leaf quality variation. Instead, this result could reflect an adaptive mechanism of *L. tristis* to synchronize egg production and the feeding phase with tender leaf resource availability to produce a new generation of beetles (class 1 type described earlier; see Sabu et al. 2008 for details) with more food reserves and better survival chances during the forthcoming dormancy phase. The eggless period towards the last phase of post-dormancy corresponds to the period of home invasion and the onset of rainfall. What leads to cessation of egg laying during this period is not understood, and it could be linked to the inherent genetic disposition towards dormancy (Denlinger 1986; Leather et al. 1995 ).

### Leaf age-related variations in rubber leaf quality

Variations in the levels of major nutrients and moisture content occur in deciduous rubber tree leaves of various ages, and the highest levels occur in tender leaves, likely due to the leaf nutrient resorption mechanism of senescing leaves ( Killingbeck 1996 ; Eckstein et al. 1998 ; Aerts and Chapin 2000 ; Van Heerwaarden et al. 2003 ). The nutrient resorption mechanism is considered one of the most important plant nutrient conservation mechanisms ( Wright and Westoby 2003 ; Yan et al. 2006 ; Huang et al. 2007 ). Foliar nutrient concentrations remain relatively constant from the time of full leaf expansion to the beginning of senescence and then decrease rapidly as foliar nutrients are resorbed prior to abscission ( Hevia et al. 1999 ). N, P, and K are mobile nutrients that are easily withdrawn from senescing tissues, and K is known for leaching ( Perry 1994 ; Lal et al. 2001 ; Hagen-Thorn et al. 2006 ). Hence, the fall in the levels of N and P in older leaves is attributed to intensive nutritive resorption, and the fall in K levels is likely due to leaching loss during the prolonged monsoon period in addition to resorption. Earlier studies suggest that Mg is moderately resorbed (up to 20%), whereas Ca is not resorbed prior to leaf abscission ( Hagen-Thorn et al. 2006 ). Ca is an immobile nutrient, leading to its higher concentration ( Epstein 1972 ; Perry 1994 ; Lal et al. 2001 ) in senescent leaves. These data indicate that rubber is a ‘‘nutrient conservative’’ species with high nutritive resorption during leaf senescence, and the senescent and dry leaves are therefore of lower nutrient quality compared to tender leaves.

### The effects of leaf age-related variations in chemical quality on the post-dormancy survival and fecundity of *L. tristis*

The present study reveals that tender leaves are essential for *L. tristis* to enter into the reproductive phase and complete its life cycle and that the levels of N and water, the two most important nutritional components for growth, are high in tender leaves; furthermore, herbivore performance (survival, growth, and reproductive capacity) was also high in tender leaves ( Mattson 1980 ; Raupp and Denno 1983 ; Osier and Lindroth 2001 ; Awmack and Leather 2002; Holton et al. 2003 ). The high correlation between N and moisture content indicates that due to the multicollinearity between the variables in the regression analysis, the N values are masking the effect of moisture content on fecundity; otherwise, the moisture content would have been a significant contributor. No broad generalizations about the impacts of other minerals on the longevity and fecundity of *L. tristis* can be made from these data, as other nutrients and defensive components of the diet modify their effects ( Martel 1998 ; Clancy 1992 ).

### Conclusions

The results of the experiments, though conducted in controlled conditions, could be used to forecast the performance of *L. tristis* in field conditions and could be included in decisions on population management and control. The present study shows that the availability of tender leaves with high levels of N and moisture is a critical factor that determines the fate of adult post-dormancy *L. tristis* beetles and the survival of the next generation. Since post-dormancy *L. tristis* obtains tender leaves from the powdery mildew-mediated premature leaf fall, the control of the premature leaf fall will reduce the population of the next generation of beetles. Additionally, these results imply that because tender leaves are essential for the reproductive maturity of *L. tristis* and are available only for a limited period of time, because wet conditions drive the beetles indoors, it is highly likely that upon removing tender leaves, *L. tristis* will remain univoltine in the rubber plantation belts.
